# Experimental-Numerical Study on the Flexural Ultimate Capacity of Prestressed Concrete Box Girders Subjected to Collision

**DOI:** 10.3390/ma15113949

**Published:** 2022-06-01

**Authors:** Yong Li, Zijie Yu, Qifan Wu, Yongqian Liu, Shang Wang

**Affiliations:** Laboratory of Roads and Railway Engineering Safety Control, Shijiazhuang Tiedao University, Shijiazhuang 050043, China; lyncdw@126.com (Y.L.); yuzijie2240170626@163.com (Z.Y.); wuqifan573@163.com (Q.W.); wangshangchrome@hotmail.com (S.W.)

**Keywords:** prestressed concrete box girder, concrete damage, reinforcement and strand fracture, ultimate flexural bearing capacity, destructive test, traffic load capacity

## Abstract

Precise evaluation for flexural ultimate capacity of bridges which are subjected to the collision of over-height trucks is essential for making decisions on corresponding maintenance, strengthening or replacement. When the span of a cross-line continuous bridge with a double-box girder was hit by an overly high vehicle, the concrete floor of one girder was severely damaged, and part of the prestressed strands and reinforcements in the girder were broken. After the double-box girder was removed and separated into two single box girders, the ultimate flexural capacity of both box girders was studied by destructive tests, and a comparison was made between the damaged and undamaged girders. Moreover, finite element analysis was conducted to simulate the failure process. The results show that the flexural bearing capacity of the damaged box girder decreased by 33%, but it was still 1.07 times greater than the design bearing capacity, which basically meets the design requirements. Also, the damaged box girder showed a desirable serviceable limit state for three-axle vehicles and five-axle vehicles, but showed an undesirable serviceable limit state for six-axle vehicles. This study shows that repairing or strengthening the damaged span may be better than demolishing and rebuilding the whole superstructure bridge.

## 1. Introduction

As a major part of road traffic, one important function of bridges is to cross existing traffic lines. If the height under bridge spans is insufficient, or over-height trucks pass under bridges, then the overcrossing superstructure of bridges may be hit by traffic loads to varying degrees [1], which may lead to concrete spalling, reinforcement and strand exposure and even fracture. Damages along prestressed concrete bridges provoke corrosion of steel reinforcements and prestressed strands and even significant prestress losses [2,3]. Different damage degrees lead to different effects on the ultimate bearing capacity of girders, which influences the corresponding maintenance, strengthening or replacement decision [4].

After bridges were built, the actual ultimate bearing capacity was always larger than the design bearing capacity. With increasing service life, the bearing capacity of bridges would decrease due to a gradual increase of the traffic load level and durability degradation [5], but it always met the requirements of the design load capacity [6]. Once damage to the bridge is serious enough to affect the bearing capacity, a quick evaluation of the bearing capacity is necessary to determine the maintenance countermeasures [7,8]. At present, the actual bearing capacity is assessed mainly according to the reduction and correction of the design bearing capacity, based on the limit state method for highway bridges in China. The actual ultimate bearing capacity can be obtained by carrying out failure tests of bridges, which are about to be torn down [9,10].

To obtain the actual bearing capacity of existing bridges [11,12], destructive tests have been carried out to provide a reference for the evaluation of the bearing capacity of bridges [13,14]. There are many existing studies on reinforced concrete bridges, such as on reinforced concrete slabs [15] and reinforced concrete beams [16,17,18]. All these studies showed that the flexural capacity obtained by destructive load tests of existing bridges was greater than that obtained by linear elastic analysis, or other assessment methods, according to nondestructive tests. However, to date, there have been few related studies on prestressed concrete bridges, especially on the actual bearing capacity of prestressed concrete box girders with serious damage.

One span of a prestressed concrete precast double-box girder of a continuous bridge was seriously damaged by the collision of an over-height truck, and it was decided to demolish the whole superstructure after the technical status assessment and bearing capacity evaluation [19]. To study the ultimate flexural bearing capacity of the prestressed concrete box girder with severe damage, destructive tests of the undamaged beam and the damaged beam were carried out separately, and the failure process of the tested beams was simulated by finite element model analysis. The actual flexural bearing capacity and traffic loading capacity of the damaged prestressed concrete box girder were evaluated, and the results provide a reference for similar maintenance, strengthening or replacement decisions to in-service prestressed concrete box girders with collision damage.

## 2. Engineering Situations

**Figure 1:**
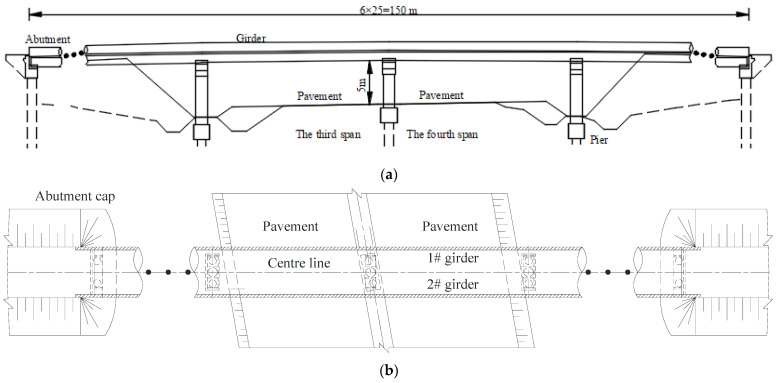

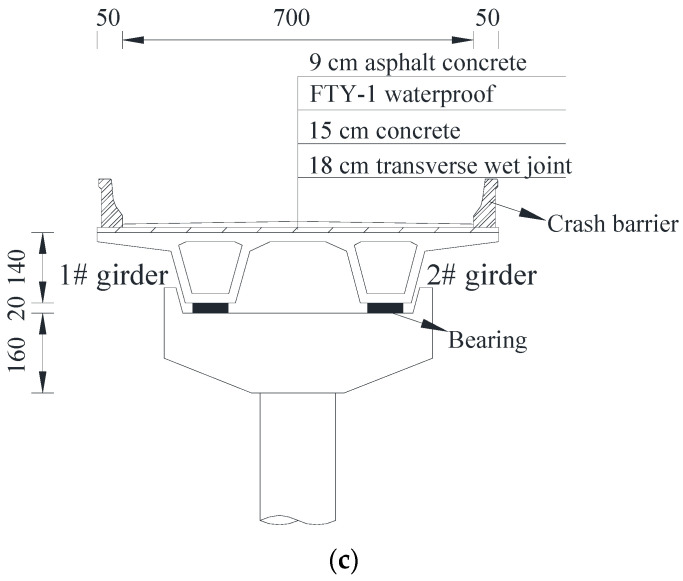
Details of the whole bridge layout (Unit: cm). (**a**) Elevation arrangement; (**b**) Plane layout; (**c**) Cross-sectional layout.

**Figure 2:**
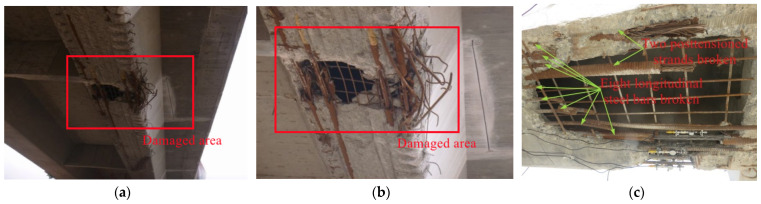
Diseases at the bottom floor of box girder #2 at the fourth span. (**a**) Damaged area; (**b**) Concrete fell off; (**c**) Posttensioned strands and longitudinal steel bars broken.

A six-span prestressed concrete continuous bridge is shown in Figure 1. The superstructure of the bridge consists of two box girders, and the bridge deck is paved with a 12 cm cast-in-place concrete leveling layer and a 9 cm asphalt concrete layer, as shown in Figure 1c. Due to the third and fourth span of the bridge crossing the traffic line, two of the double-box girders were seriously damaged after a collision with a large truck, as shown in Figure 2. Considering the safety of bridge service, the whole girder of this bridge was removed after only being in service for six years.

**Figure 3:**
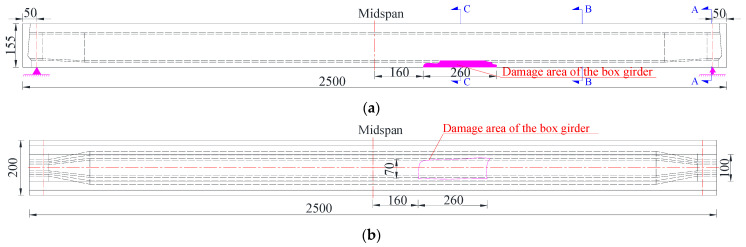
Damaged area of damaged girder #2 at the fourth span (Unit: cm). (**a**) Front view; (**b**) Bottom view.

As shown in Figure 2, the significant diseases of prestressed concrete box girder #2 were as follows. A total of 1.82 m^2^ of the concrete bottom plate close to the midspan fell off due to the collision, two posttensioned strands and eight longitudinal steel bars in the bottom plate were broken, and the unbroken strands and reinforcements in the damaged area were exposed to air. The damaged area of damaged girder #2 is shown in Figure 3. Correspondingly, undamaged box girder #1 was in good overall condition with only slight abrasion in the local position.

**Figure 4:**
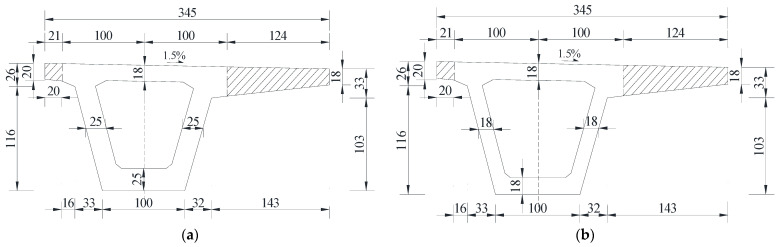
Cutting diagram of the box girder at the fourth span (Unit: cm). (**a**) A-A section; (**b**) B-B section.

To facilitate easy transport of the demolished girders and carry out failure tests, the flanges of the demolished box girders were cut locally, as shown in Figure 4. To ensure that the failure tests proceeded smoothly, a simply supported system at the girder end was adopted for the demolished box girders.

**Figure 5:**
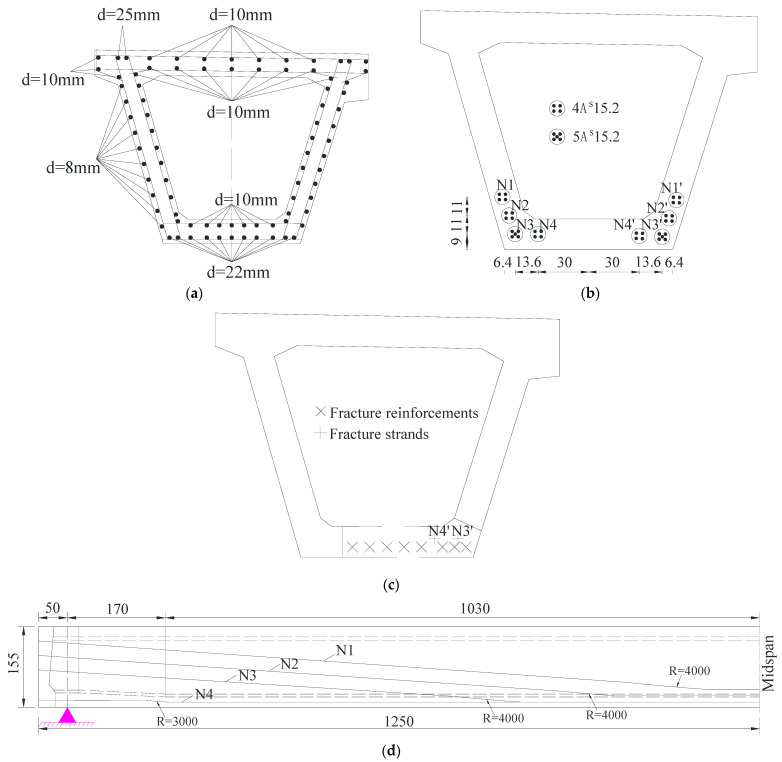
Reinforcements and strands arrangement in the girder (Unit: cm). (**a**) Steel bars in the undamaged beam (B-B); (**b**) Prestressed strands in the undamaged beam (B-B); (**c**) Steel bar condition at the damaged region of box girder #2 (C-C); (**d**) Strand arrangement.

The arrangement of strands and steel bars in the box girder is shown in Figure 5, and the strength parameters of the concrete, strands and steel bars are shown in Table 1.

## 3. Failure Test of the Undamaged Girder

### 3.1. Calculation of the Flexural Capacity of the Undamaged Box Girder

**Figure 6:**
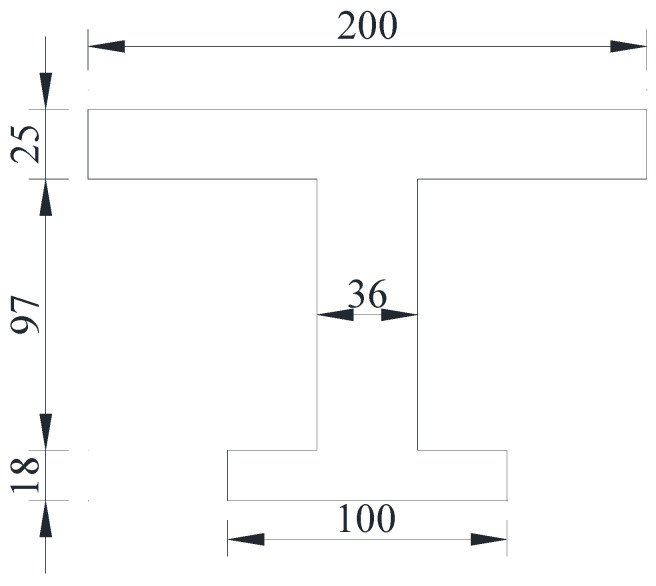
Equaled I-shape of the undamaged girder (Unit: cm).

According to the design principle of the limit state method [20], the box section can be equaled to an I-shape when the flexural bearing capacity is calculated, as shown in Figure 6. The calculation parameters of the undamaged girder are shown in Table 2. It was concluded that the midspan section of the undamaged girder meets Equation (1), and the flexural capacity *M*_u_ was calculated according to Equation (2). The flexural bearing capacity *M*_u_ at the midspan section of the undamaged box girder was 8442 kN·m, and the cracking bending moment was approximately 6984 kN·m.

(1) $$f_{\mathrm{sd}}A_{s}+f_{\mathrm{pd}}A_{p}\leq f_{\mathrm{cd}}b_{f}^{\prime }h_{f}^{\prime }+f_{\mathrm{sd}}^{\prime }A_{s}^{\prime }$$

(2) $$M_{u}=f_{cd}b_{f}^{\prime }x(h_{0}-\frac{x}{2})+f_{sd}^{\prime }A_{s}^{\prime }(h_{0}-a_{s}^{\prime })$$

where *f*_sd_ and *f*′_sd_ are design tensile strength and compressive strength of longitudinal reinforcements, *f*_pd_ is design tensile strength of strands and *f*_cd_ is design compressive strength of concrete. *A*_s_ and *A*′_s_ are areas of longitudinal reinforcement. *A*_p_ is section area stands. *h*′_f_ and *b*′_f_ are thickness and width of flanges for the equaled I-shape and *h*_0_ is the effective height, while *x* is the height of the compressive zone. Finally, *a*′_s_ is the distance between the area centroid of compression reinforcement and concrete edge.

### 3.2. Loading Scheme

**Figure 7:**
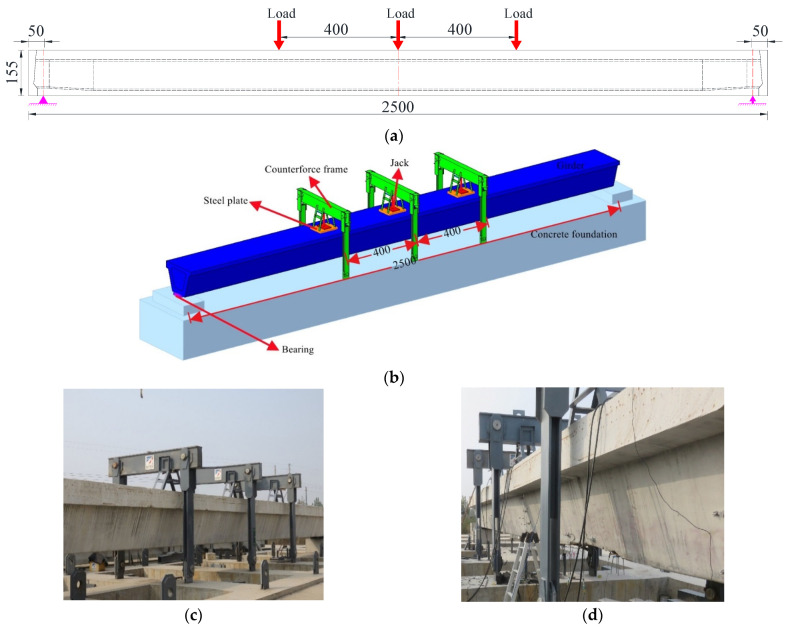
Destructive loading layout (Unit: cm). (**a**) Loading location; (**b**) Schematic loading arrangement; (**c**) Right view of field loading system; (**d**) Left view of field loading system.

A reaction frame assembled by slot steel was adopted in the loading device. The destructive test was loaded by three hydraulic jacks with a maximum output of 2000 kN, as shown in Figure 7a. Steel plates with specifications were set between the jacks and the girder. Pressure sensors with a range of 0–2000 kN were installed between the hydraulic jacks and the reaction frame, which was assembled by slot steel components. Bearings were removed from the bridge and placed under the bottom plate of the box girder and the computed span between bearings was 24 m. The test load and the test loading rate were controlled by the measured values of the pressure sensors. The test arrangement of the box girder is shown in Figure 7.

To study the failure process of the undamaged box girder, the first load, that was 70% of the predicted value of the cracking load, was applied and then unloaded. The second load, that was 85% of the predicted value of the ultimate load, was utilized and then unloaded, and the third load was applied until the box girder failed. During each loading process, each stage was loaded with 50 kN. When the load of each jack exceeded 450 kN, displacement control was adopted for loading until the box girder was destroyed.

**Figure 8:**
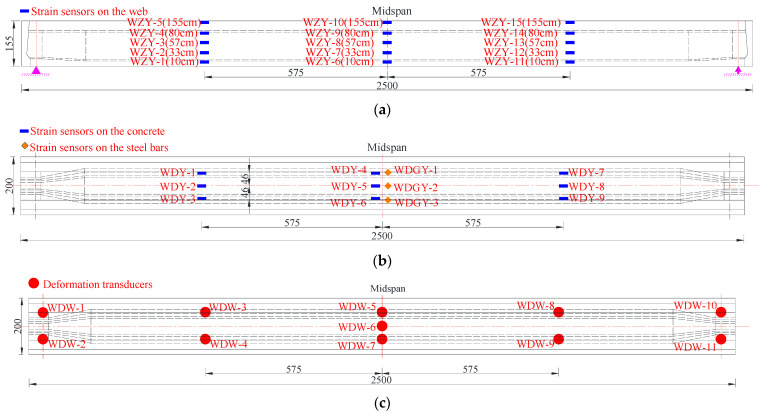
Sensor arrangement for the destructive test of the undamaged box girder (Unit: cm). (**a**) Strain sensor arrangement at the concrete web; (**b**) Strain sensor arrangement at the concrete floor and steel bars; (**c**) Deformation sensor arrangement at the bottom of the girder.

Strain and deformation measuring points were laid at the critical section of the undamaged box girder. The specific positions and labels of strain and deformation measurement points are shown in Figure 8. Prior to installing the strain gauges of the reinforcements, the concrete cover at the installation positions was removed first, and then the reinforcement surface was polished until smooth. During the test process, all kinds of sensor data were monitored and collected. The equipment used in the test is shown in Table 3.

### 3.3. Establishment of the Refined Model

**Figure 9:**
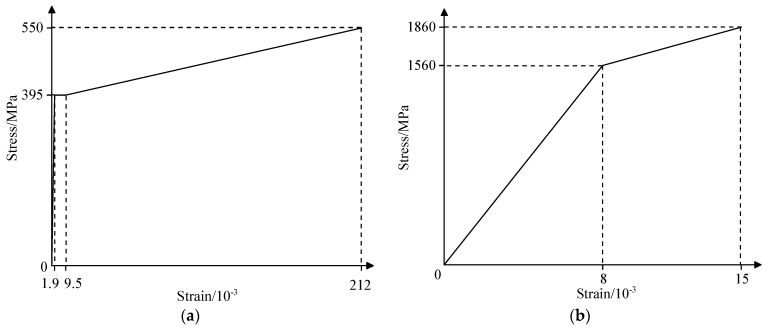

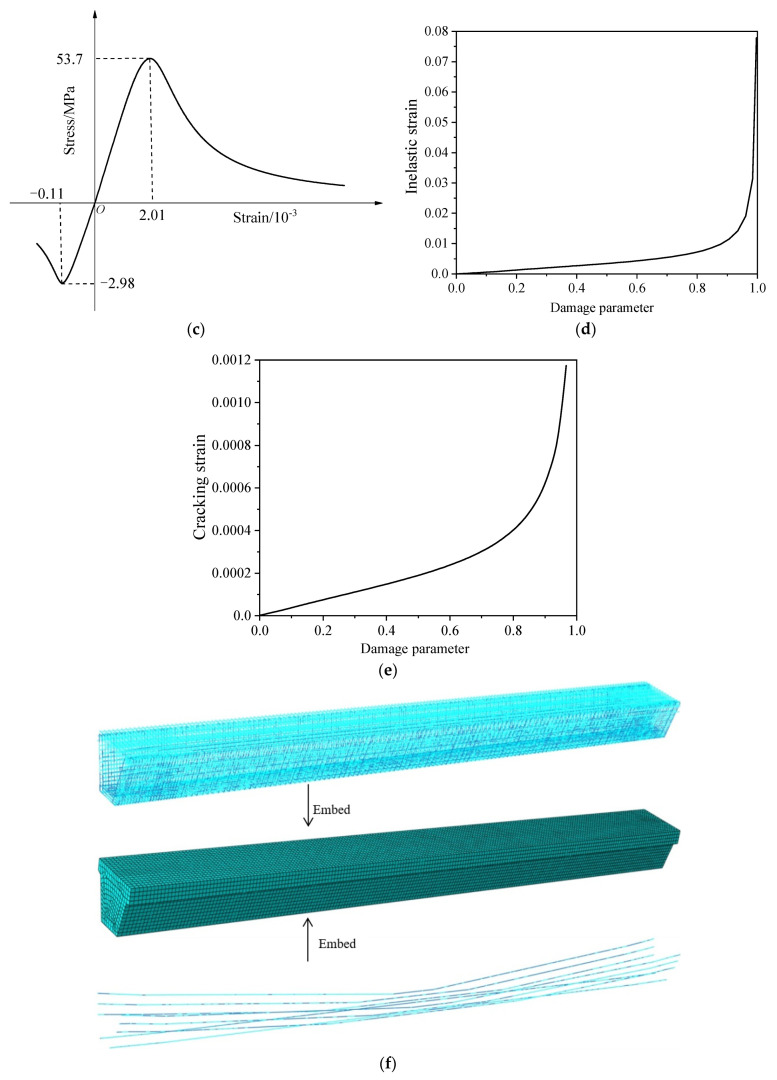
The refined finite element model of the box girder. (**a**) Stress vs. strain curve of reinforcements; (**b**) Stress vs. strain curve of pretensioned strands; (**c**) Stress vs. strain curve of concrete; (**d**) Compression damage of concrete; (**e**) Tension damage of concrete; (**f**) Finite element model.

The finite element model of the box girder was established in ABAQUS to predict the destructive process and the ultimate bearing capacity [21]. A concrete damaged plasticity model was adopted to simulate the behavior of the concrete, and the stress-strain relationships of the compression behavior and tensile behavior were selected, according to the Chinese design code for concrete structures [22]. The trilinear model was used for the constitutive model of reinforcements that have distinct yielding stages in the tensile stress-strain curve, while the bilinear model was adopted to simulate the behavior of pretensioned strands without an obvious yielding stage. The details in the stress-strain curve of reinforcements and prestressed strands are shown in Table 4, and the nominal stress, which was obtained according to the test data, was converted into true stress and input into the finite element model. The stress-strain curves and damage curves for concrete used in the finite element model are shown in Figure 9a–e.

Eight-node hexahedral reduction integral element C3D8R was used for concrete, and the truss element was used for reinforcements and prestressed strands, as shown in Table 5. All the reinforcement and strand elements were embedded into the concrete region, as shown in Figure 9f. Since the actual effective prestress of the box girder cannot be measured, to consider the prestress loss caused by friction loss, stress relaxation and shrinkage, and creep of concrete, the effective prestress, after considering the loss, was calculated according to the current design code of China [23]. The effective prestress was calculated according to Equations (3)–(9), and the specific results are shown in Table 6. Defining the linear expansion coefficient of strands and applying a cooling temperature load can lead to the shrinkage of strands to simulate the effective prestress. The tension control stress of the strands was 1395 MPa, and the effective prestress of the N1, N2 and N3 strands was 1113.5 MPa, and that of N4 strand was 1154.8 MPa, after considering the prestress loss. Surface binding constraints were set between the steel plates and the girder, and between the bearings and the girder. The reference points were set at the top of each steel plate and used as the loading points. Coupling constraints were adopted between the reference points and the steel plates.

(3) $$\sigma_{\mathrm{pe}}=\sigma_{\mathrm{con}}-\sigma_{l1}-\sigma_{l2}-\sigma_{l4}-\sigma_{l5}-\sigma_{l6}$$

where $\sigma_{l1}$ is frictional losses, $\sigma_{l2}$ is the anchorage losses, $\sigma_{l4}$ is the prestress loss caused by elastic compression of the concrete, $\sigma_{l5}$ is the prestress loss caused by relaxation of prestressed strands and $\sigma_{l6}$ is the time-dependent loss caused by creep and shrinkage of the concrete.

(4) $$\sigma_{l1}=\sigma_{\mathrm{con}}\left[1-e^{-\left(\mu \theta +kx\right)}\right]$$

where *μ* is the coefficient of friction, with value 0.25, and *θ* is the angle of tangent of curve pipes. *K* is the influence coefficient of friction, with value 0.0015, and *x* is the Length of pipe from the tensioning end to the calculated section.

(5) $$\sigma_{l2}=\Delta \sigma \frac{l_{f}-x}{l_{f}}$$

(6) $$\Delta \sigma =2\Delta \sigma_{d}l_{f}$$

where $\Delta \sigma_{d}$ is the prestress loss per unit length caused by pipeline friction, Δσ is the prestress loss of prestressed reinforcement under the tension end anchor, after considering reverse friction, $l_{f}$ is the influence length of kickback-friction, and $l_{}$ is the distance from tensile end to anchorage end, and the value is 12.2 m.

(7) $$\sigma_{l4}=\alpha_{\mathrm{EP}}\sum \Delta \sigma_{\mathrm{pc}}$$

where $\alpha_{\mathrm{EP}}$ is the ratio of elastic modulus of prestressed strands to elastic modulus of concrete, with value 5.65, and $\Delta \sigma_{\mathrm{pc}}$ is the concrete stress at the gravity center of pre-tensioned strands caused by post-tensioned strands.

(8) $$\sigma_{l5}=\Psi \cdot \zeta \left(0.52\frac{\sigma_{\mathrm{pe}}}{f_{\mathrm{pk}}}-0.26\right)\sigma_{\mathrm{pe}}$$

where Ψ is the tension coefficient, with value 0.9, ζ is the relaxation coefficient of steel bar, with value 0.3, and $\sigma_{\mathrm{pe}}$ is the effective stress of strands after anchoring; $\sigma_{\mathrm{pe}}=\sigma_{\mathrm{con}}-\sigma_{l1}-\sigma_{l2}-\sigma_{l4}$.

(9) $$\sigma_{l6}(t)=\frac{0.9\left[E_{P}\varepsilon_{\mathrm{cs}}\left(t,t_{0}\right)+\alpha_{\mathrm{EP}}\sigma_{\mathrm{pc}}\phi \left(t,t_{0}\right)\right]}{1+15\rho \rho_{\mathrm{ps}}}$$

where $E_{P}$ is the elastic modulus of prestressed strands, and the value is 1.95 × 10^5^ MPa, ρ is the reinforcement ratio of longitudinal reinforcement in tension area, and $\rho_{\mathrm{ps}}$ is the reinforcement ratio of strands and ordinary reinforcement in tension area. $\varepsilon_{\mathrm{cs}}\left(t,t_{0}\right)$ is the shrinkage strain of concrete when the anchorage age of prestressed steel bar is $t_{0}$ and the calculation age is t. $\phi \left(t,t_{0}\right)$ is the creep coefficient when the anchorage age of prestressed steel bar is $t_{0}$ and the calculation age is t.

### 3.4. Test Results

**Figure 10:**
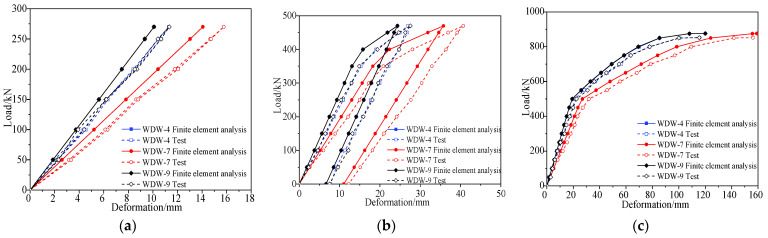
Deformation vs. load of the undamaged box girder. (**a**) First cycle of loading and unloading; (**b**) Second cycle of loading and unloading; (**c**) Third loading to failure.

**Figure 11:**
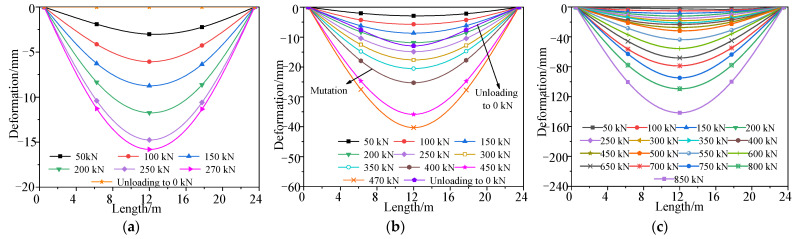
Deformation curve of the undamaged test box girder bottom plate. (**a**) First cycle of loading and unloading; (**b**) Second cycle of loading and unloading; (**c**) Third loading to failure.

A comparison of the midspan deformation of the undamaged box girder in each cyclic loading, between the measured results and theoretical prediction, is shown in Figure 10, and the deformation distribution along the longitudinal direction of the box girder, measured in different loading stages, is shown in Figure 11. Figure 10 shows that the refined finite element analysis results are close to the test results, and the failure process of the undamaged box girder contains an elastic stage, a working stage with cracks and a destructive stage after the yield of the steel bars. In the elastic stage, the deformation of the box girder was about 2.9 mm in each stage loaded with 50 kN. When the load of each loading point was added to 362 kN, cracks at the box girder bottom appeared with a width of 0.16 mm, and the bending moment of the box girder was 7034 kN·m in the midspan. The steel bars began to yield when the load increased to 801 kN. In the final failure, the concrete on the top of the box girder was crushed, the maximum crack width of the box girder was 2.03 mm and the maximum deformation was 142 mm when the load increased to 850 kN. Figure 11 shows that the bending deformation of the girder maintained good symmetry under the condition of symmetric loading.

**Figure 12:**
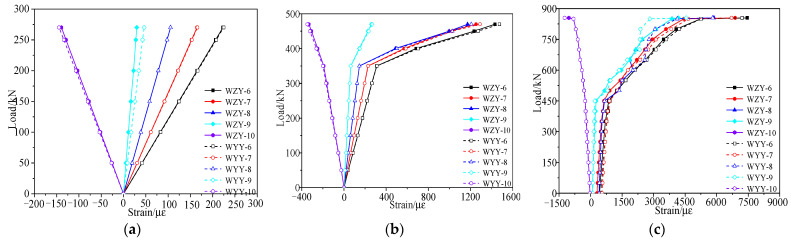
Strain vs. load curve of the concrete web at midspan of the undamaged test box girder. (**a**) Elastic stage; (**b**) Working stage with cracks; (**c**) The failure stage.

**Figure 13:**
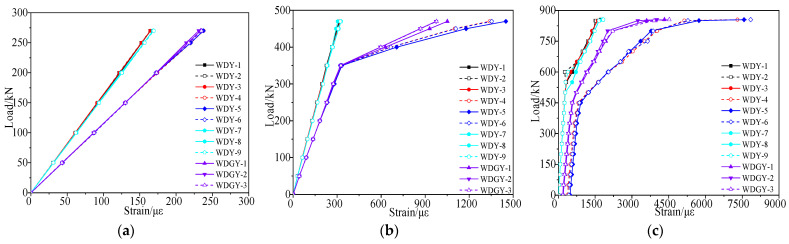
Strain vs. load curve of the undamaged test box girder bottom plate. (**a**) Elastic stage; (**b**) Working stage with cracks; (**c**) The failure stage.

According to the failure test, the strain vs. load curves under each load stage were obtained, as shown in Figure 12 and Figure 13. Figure 12 shows that the inflection points of the strain vs. load curves appeared due to cracks when the load reached 362 kN, and the concrete strain of the right web was slightly larger than that of the left web at the same load stage. Since the section of the test box girder was not completely symmetric, the height of the left web was larger than that of the right web. Figure 13 shows that the strain of the undamaged box girder showed an obvious three-stage development process when it was loaded to failure in the third stage, with the reinforcements yield last.

### 3.5. Ultimate Flexural Capacity of the Undamaged Box Girder

The actual ultimate bending capacity can be deduced according the summation of the bending moment, due to the combination of the actual failure load and the undamaged box girder’s own weight. The actual ultimate bending capacity was compared with the theoretical value and the predicted value, and the ratio was obtained by comparing experimental value and predicted value with the theoretical value, as shown in Table 7. As seen from Table 7, the refinement finite element analysis results of the undamaged prestressed concrete box girder were close to those obtained from the destructive test, which were both greater than the theoretical bearing capacity. The cracking moment of the theoretical value and the predicted value were close to those obtained from the destructive test. The actual bearing capacity of the undamaged box girder was approximately 1.60 times that of the theoretical value, which was calculated according to the design strength, indicating that the undamaged box girder had a larger safety reserve. The maximum bending moment, due to maximum loading combination of the single girder of the bridge, was 5001 kN·m, so the bearing capacity of the undamaged box girder was approximately 2.7 times the maximum load effect of the design.

## 4. Failure Test of the Damaged Girder

### 4.1. Calculation of the Flexural Capacity of the Damaged Box Girder

**Figure 14:**
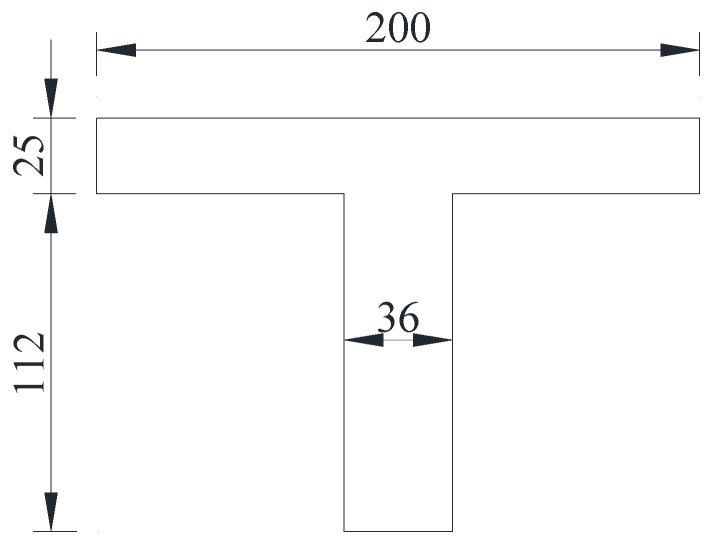
Equaled T-shape of the damaged girder (Unit: cm).

According to the design principle of the limit state method [20] and the diseases of the damaged girder, the section is equaled into the T-shape, as shown in Figure 14, and the calculation parameters of the flexural capacity of the damaged box girder are shown in Table 8. The flexural bearing capacity at the midspan section of the damaged box girder was 5472 kN·m according to Equations (1) and (2).

### 4.2. Loading Scheme

**Figure 15:**
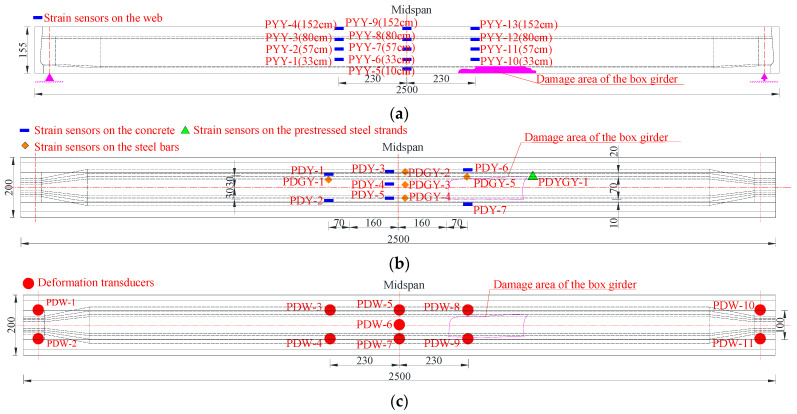
Sensor arrangement for the loading test of the damaged box girder (Unit: cm). (**a**) Strain sensors arranged on the web; (**b**) Strain sensors layout diagram of bottom plate; (**c**) Deformation sensors layout diagram of bottom plate.

The device for the destructive test of the damaged box girder was the same as that for the undamaged box girder. Strain sensors were arranged on several reinforcements and prestressed steel strands exposed in the damaged region of the bottom plate, as shown in Figure 15.

To study the failure process of the test box girder, the first load, which was 85% of the calculated value of ultimate load, was applied and then unloaded, and the second load was applied until the box girder failed. During each loading process, each stage was loaded with 50 kN. When the load of each jack exceeded 450 kN, displacement control was adopted for loading until the box girder was destroyed.

### 4.3. Establishment of the Refined Finite Element Model

**Figure 16:**
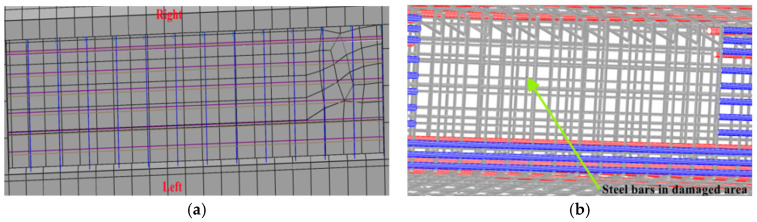
Details in the damage region of the box girder. (**a**) Concrete fell off; (**b**) Posttensioned strands and longitudinal steel bars broken.

The corresponding elements of each component in the damaged region at the bottom plate of the damaged box girder could be deactivated in the finite element model to simulate concrete falling, reinforcement corrosion and strand breakage. The components of concrete, reinforcements and strands were segmented according to the actual damage shedding and fracture location, and the segmented parts were established into a set, and then passivated in the analysis step. Reinforcement corrosion was simulated by reducing the yield strength and the cross-sectional area according to the corrosion rate. The interaction between exposed reinforcements and concrete was not considered. In order to ensure the normal operation of the finite element analysis, it was necessary to expose the reinforcement parts and concrete sharing at the same nodes at the junctions, which ensured that the reinforcement truss elements were fully embedded in the concrete elements. The details of the damaged region of the damaged box girder are shown in Figure 16.

### 4.4. Test Results

**Figure 17:**
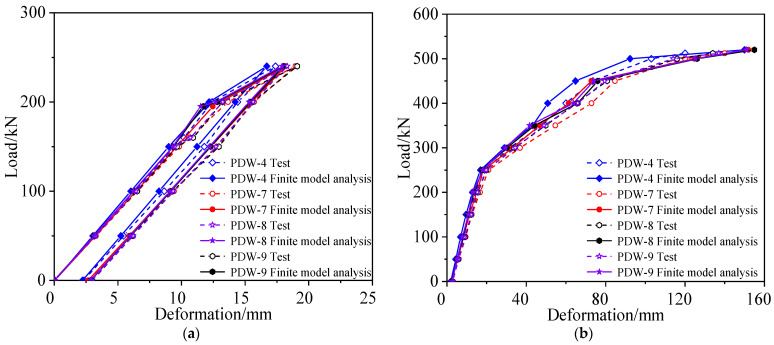
Deformation vs. load of the damaged box girder. (**a**) First cycle of loading and unloading; (**b**) Second loading to failure.

**Figure 18:**
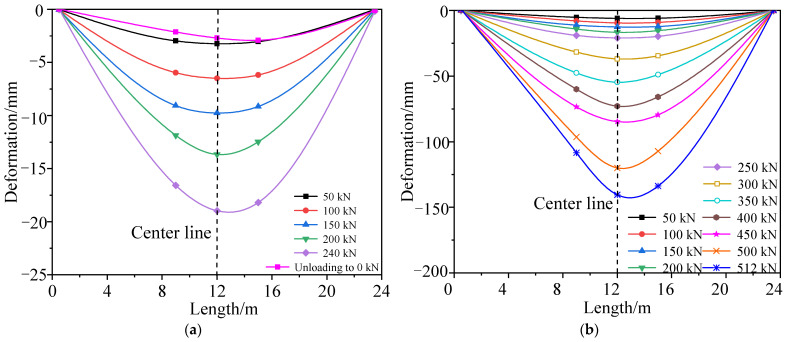
Deformation curve of the damaged test box girder bottom plate. (**a**) First cycle of loading and unloading; (**b**) Second loading to failure.

A comparison of the midspan deformation of the damaged box girder in each cyclic loading between the measured results and theoretical analysis is shown in Figure 17, and the deformation distribution along the longitudinal direction of the damaged box girder measured in different loading stages is shown in Figure 18. Figure 17 shows that the refined finite element analysis results were close to the failure test results, after considering the loss of concrete and the fracture of steel bars in the damaged area, which can reflect the actual stress state of the damaged prestressed concrete box girder. The steel bars of the bottom plate began to yield when the load was 427 kN. Compared with the undamaged box girder, the bar yield load reduced by about 47%, and the damaged box girder was seriously damaged but still had a certain bearing capacity, and the failure load was reduced by approximately 40% to approximately 512 kN. In the final failure, the maximum deformation was 140 mm and maximum crack width of the box girder was 2.09 mm. Figure 18 shows that the deformation curve of the box girder in the loading process was not symmetrical, and the deformation at the damaged area was larger and close to the midspan deformation, because the damaged area was close to the midspan and showed a weak stiffness.

**Figure 19:**
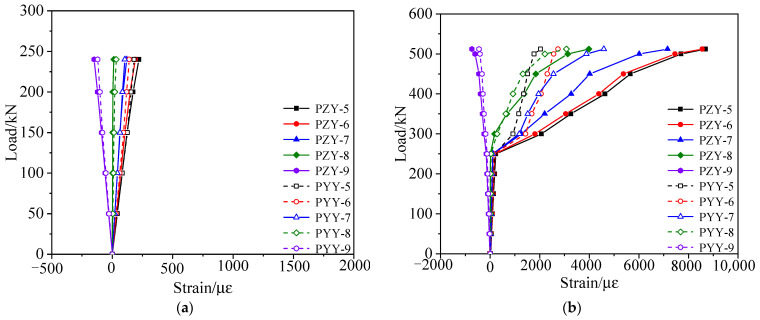
Strain vs. load curve of the concrete web at midspan of the damaged test box girder. (**a**) Working stage with cracks; (**b**) The failure stage.

**Figure 20:**
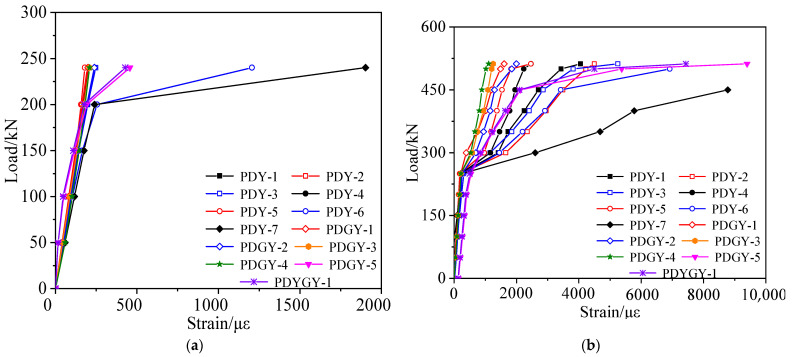
Strain vs. load curve of the damaged test box girder bottom plate. (**a**) Working stage with cracks; (**b**) The failure stage.

According to the failure test, the strain vs. load data under each load cycle could be obtained, as shown in Figure 19 and Figure 20. Figure 19 and Figure 20 show that the strain of the concrete and steel bars in the damaged area and its vicinity was large, while the strain of other undamaged areas was small. The reason was the lack of concrete in the damaged area and the fracture of steel bars, resulting in local stiffness weakening.

### 4.5. Ultimate Flexural Capacity of the Damaged Box Girder

The actual ultimate bending capacity can be deduced according the summation of the bending moment due to the actual failure load and the undamaged box girder’s own weight and compared with the theoretical value and the predicted value, as shown in Table 9. As seen from Table 9, the theoretical value calculated by design strength of the damaged box girder was 60.1% of the actual ultimate bending capacity obtained by the destructive test, but it was still 1.09 times of the maximum load effect of the design, which could still meet the requirements. Although the predicted value obtained by the fine finite element analysis of the undamaged prestressed concrete box girder was close to the actual ultimate bending capacity, there was still an error of 7%. Compared with the undamaged box girder, the actual bearing capacity of the damaged box girder was reduced by approximately 33%, but it was still 1.67 times greater than the most adverse load effect of the design.

## 5. Comparative Analysis

### 5.1. Flexural Stiffness

**Figure 21:**
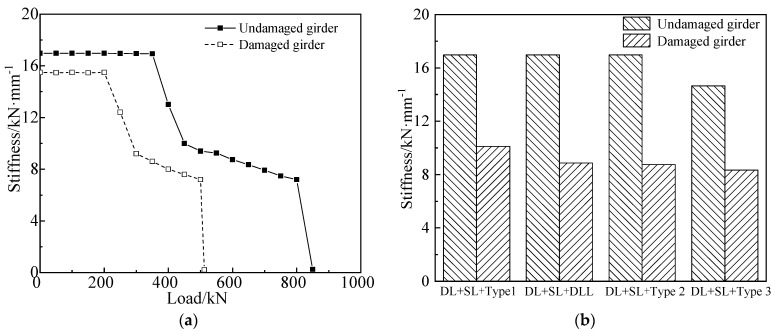
Stiffness comparison due to the destructive tests. (**a**) Load vs. stiffness degradation curve; (**b**) Stiffness degradation under different live loads.

**Figure 22:**
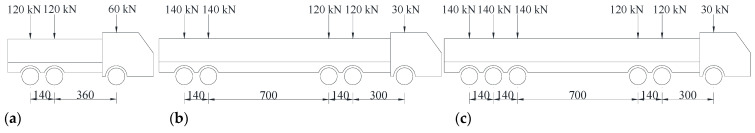

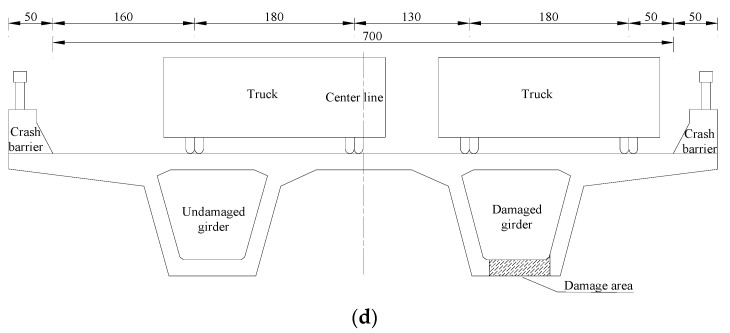
Different types of trucks and transverse layout (Unit: cm). (**a**) Type 1; (**b**) Type 2; (**c**) Type 3; (**d**) Transverse layout.

The load vs. stiffness curves of both girders, according to the failure test, are shown in Figure 21. As seen from Figure 21, DL represents the dead load, SL represents the secondary load, DLL represents the design live load, and types 1, 2 and 3 respectively represent typical three-axle vehicle, five-axle vehicle and six-axle vehicle. The three types of vehicles and transverse layout are shown in Figure 22. In Figure 21, the flexural stiffness of the girders decreased significantly when the undamaged and damaged girders were loaded to 362 kN and 201 kN, respectively, due to the appearance of new cracks. During the loading stage with cracks, the stiffness of the girders decreased slowly because of the development of cracks. The stiffness decreased swiftly when the undamaged and damaged girders were loaded to 801 kN and 427 kN, respectively, because the steel bars yielded. Compared with the undamaged girder, the stiffness of the damaged girder was reduced by 8.7% in the elastic stage and 42.7% when subjected to the load combination of dead load, secondary loads and six-axle truck load, because the damaged girder had expressed concrete loss and cracks after being stroked by trucks.

### 5.2. Flexural Deformation

**Figure 23:**
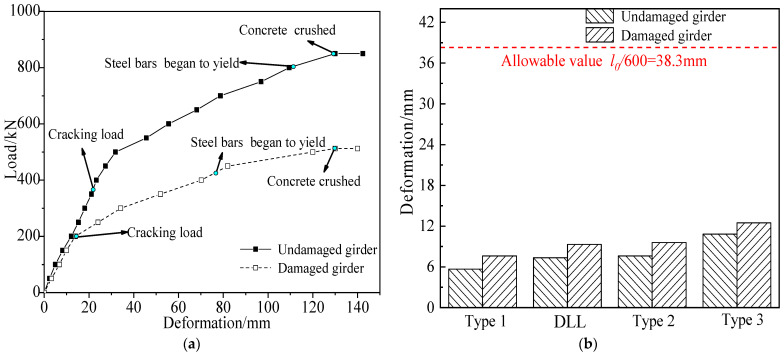
Midspan deformation comparison due to the destructive tests (**a**) Load vs. deformation curve; (**b**) Midspan deformation under different live loads.

The load vs. midspan deformation curves of both girders, according to the destructive test, are shown in Figure 23. The midspan deformations of the undamaged girder were 7.6 mm and 10.8 mm when subjected to 0.7 times the load of five-axle and six-axle vehicles. The ratios of deformation to span were 1/3158 and 1/2222, respectively; which met the allowable value of 1/600 of the specification. For the damaged girder, the midspan deformations were 9.6 mm and 12.5 mm, when subjected to 0.7 times the load of five-axle and six-axle vehicles, and the ratios of deformation to span were 1/2500 and 1/1920, respectively; which were increased by 26.1% and 15.7% compared with that of the undamaged girder, and still met the allowable value of specification. The damaged girder had expressed concrete loss and cracks after being stroked by trucks, resulting in reduction of the stiffness of the overall section. It can be concluded that the damaged girder still had a certain safety reserve of bending stiffness under normal live loads.

### 5.3. Stress

**Figure 24:**
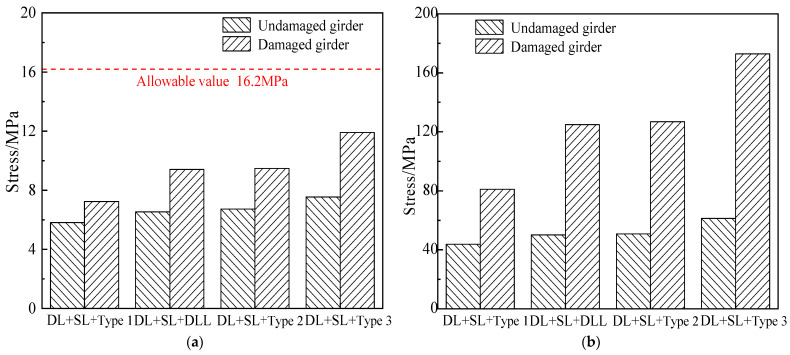
Stress comparison due to the destructive tests (**a**) Stress of concrete; (**b**) Stress of longitudinal steel bars.

The load vs. stress curve of the undamaged and damaged girders subjected to different load combinations were obtained according to the failure tests, as shown in Figure 24. In Figure 24, the concrete stress of the undamaged girder was about 7.6 MPa, which met the allowable value of 16.2 MPa of the specification subjected to the load combination of dead load, secondary loads and six-axle truck load. Stress of the damaged girder increased by about 4.3 MPa, which was still less than the allowable value 16.2 MPa of the specification. For the damaged girder, the tensile stresses of the steel bars were 126.8 MPa and 172.8 MPa, which had increased by 76 MPa and 111.5 MPa, compared with that of the undamaged girder subjected to the 0.7 times the load of five-axle and six-axle vehicles. Compared with the undamaged girder, the reason for the increase of the stress of the steel bars and concrete stress of the damaged girder was the reduction of the cross-sectional area of the reinforcement and concrete, which resulted in reduction of the stiffness of the overall section, and greater stress being brought to bear on the remaining reinforcement and concrete.

### 5.4. Crack Width

**Figure 25:**
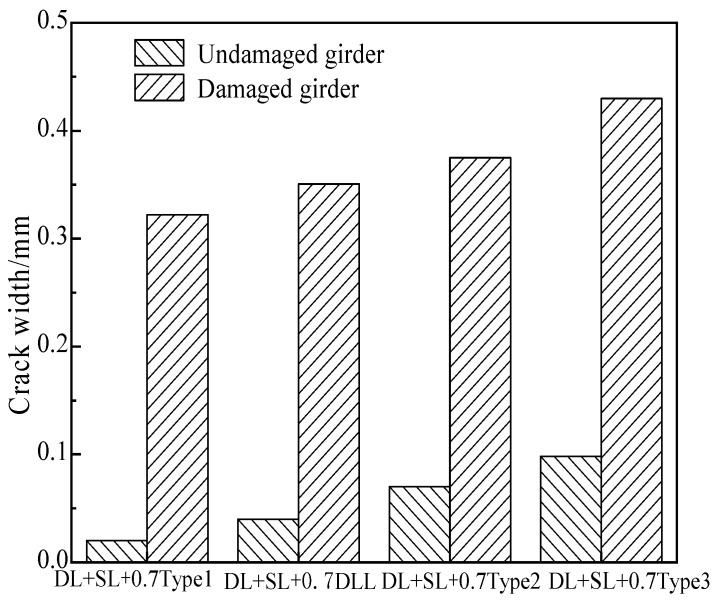
Crack width comparison due to the destructive tests.

According to the destructive test, the diagram of load vs. crack width was obtained, as shown in Figure 25. Under the load combination of dead load, secondary load and 0.7 times of six-axle truck load, the maximum crack width of the undamaged girder was 0.10 mm, because the undamaged girder bore a greater load after the defect of the damaged girder, which led to the slight crack at the bottom of the girder. For the damaged girder, the maximum crack width was 0.43 mm. Due to partial strands and steel bars being broken, the reinforcement ratio of the girder decreased from 0.33% to 0.26%, and the prestressing degree decreased from 1.35 to 0.97.

### 5.5. Traffic Load Capacity

This bridge was designed with two lanes for transportation. To verify the capacity of the bridge under the state of damage, three different types of standard vehicles, including a 3-axle truck, 5-axle truck and 6-axle truck, were considered to pass on the bridge to obtain the combined effect of the maximum bending moment in the damaged span of the box girder, as shown in Figure 22a–c. The transverse layout of trucks is shown in Figure 22d. Then, the combination of action effects was compared with the theoretical and tested values of the flexural bearing capacity to obtain the actual safety coefficient for each type of truck, as shown in Table 10.

As seen from Table 10, the undamaged prestressed concrete box girder had a significant bearing capacity, and the flexural ultimate bearing capacity obtained through the destructive test was approximately 60% higher than the design bearing capacity obtained by the limit state method. For the damaged prestressed concrete box girder, the bearing capacity did not meet the traffic requirements through theoretical analysis because of serious damage and disease. However, the actual bearing capacity obtained through the damage test did actually meet the traffic requirements. The theoretical bearing capacity was calculated according to the design value of material strength, less than the actual material strength tested. It was more reasonable to use external prestressing technology to reinforce the damaged span than to remove and replace the whole bridge.

## 6. Conclusions

A prestressed concrete continuous girder bridge was demolished due to serious damage caused by vehicle pounding. Through the damage test of dismantled box girders, the flexural ultimate bearing capacity of the undamaged box girder and the damaged box girder were compared and analyzed, and the following conclusions drawn.

(1) Due to the pounding between a truck and the bridge, the bottom plate of the box girder was damaged, and several reinforcements and pretensioned strands were broken, which decreased the ultimate bearing capacity of the box girder. According to the limit state method, the theoretical bearing capacity of the damaged box girder was reduced by approximately 64%, which did not meet the operational requirements of the bridge and led to the demolition and replacement of the whole superstructure of the bridge.
(2) The ultimate flexural bearing capacity of the undamaged and damaged box girders was obtained by destructive tests. It was proven that the ultimate bearing capacity of the undamaged box girder was distinctly higher than the theoretical results. Although the actual bearing capacity of the damaged box girder was reduced by approximately 33%, it could still meet the operational requirements, which means that the residual reinforcements and stands still provided considerable flexural loading capacity of the box girder.
(3) Through the comparative analysis of damaged and undamaged box girders, the results showed that, compared with the undamaged box girder, the stiffness of the damaged box girder was reduced by 42.7% under the load combination of dead load, secondary loads and six-axle truck load, but the stress of concrete, the stress of steel bar, deformation and crack width were increased by 56.5%, 55%, 15.7%, 330%, respectively. Among these, the stress and deflection still met the requirements of the design specification. However, due to the partial strands and steel bars of the girder being broken by the reduction of the reinforcement rate and the degree of prestressing, converted into a type B prestressed concrete member, the result was an increase of crack width, which did not meet the requirements of the design specification.
(4) Considering the measured material properties and the degree of damage, refinement finite element analysis resulted in a more credible bearing capacity than the theoretical analysis result. As the original superstructure of the bridge was a fabricated double-box girder, continuous, bridge, although one girder had serious damage, it still had the capacity to meet design requirements, because the transverse connection was in good condition. Repairing or strengthening the damaged span would have been more economic and reasonable than demolition and reconstruction of the whole superstructure of the bridge. However, there was no failure test verification for the repaired and strengthened girder, so a comparative failure test study will be conducted on the repaired and strengthened beam and the undamaged beams in the future.

## Figures and Tables

**Table 1:** Material parameters.

Material	Type	Characteristics Value/MPa		Design Value/MPa		Measured Actual Value/MPa	
		Compressive Strength	Tensile Strength	Compressive Strength	Tensile Strength	Compressive Strength	Tensile Strength
C50	/	32.4	2.65	22.4	1.83	55.7	/
HRB335	d = 22 mm	335	335	280	280	/	382
R235	d = 10 mm	235	235	195	195	/	296
15 mm strand	7-wirestrand	/	1860	390	1320	/	1920

**Table 2:** Material and parameters of the undamaged box girder.

*ƒ*_cd_/MPa	*ƒ*_sd_/MPa	*ƒ*′_sd_/MPa	*ƒ*_pd_/MPa	*A*_s_/mm^2^	*A*′_s_/mm^2^	*A*_p_/mm^2^	*h*_0_/mm	*a*′_s_/mm	*x*/mm
22.4	280	280	1260	4181	2670	4726	1253	45	142.4

**Table 3:** Test equipment.

Type	Precision	Amount	Function
HY-65B3000B strain sensor	0.1 με	50	Measuring the strain of concrete
HY strain acquisition system	/	1	Collecting strain readings
HY-65050P deformation transducer	0.001 mm	15	Measuring deformation
HY65R100 deformation transducer	0.001 mm	5	Measuring deformation
HY65R010 deformation transducer	0.01 mm	8	Measuring crack width
Strain sensor	0.1 με	40	Measuring the strain of steel bars
DH3815N strain acquisition system	/	1	Collecting strain readings
Hydraulic jack	200 T	3	Outputting pressure load
JMZX-3415 pressure sensor	0.1 kN	3	Measuring pressure load
JMZX-3006 comprehensive tester	/	3	Collecting readings of pressure load
DH5922N bridge dynamic tester	0.001 HZ	1	Collecting readings of fundamental frequency
Steel inspector	1 mm	1	Scanning to determine the position of reinforcement and strand

**Table 4:** Details in the stress-strain curves of reinforcements and prestressed strands.

Type	Yield Strength/MPa		Ultimate Tensile Strength/MPa	
	Nominal Stress	True Stress	Nominal Stress	True Stress
Steel bar	335	395	466	550
Prestressed strand	1322	1560	1576	1860

**Table 5:** Details of the finite element model.

Model	Unit Type	Mesh Amount	Mesh Size/mm
Concrete box girder	C3D8R	20,533	120
Steel bar	Truss	18,065	240
Prestressed strand	Truss	810	240

**Table 6:** Calculation of prestress loss.

Number of Strands	$\sigma_{\mathrm{con}}$ /MPa	$\sigma_{l1}$ /MPa	$\sigma_{l2}$ /MPa	$\sigma_{l4}$ /MPa	$\sigma_{l5}$ /MPa	$\sigma_{l6}$ /MPa	$\sigma_{\mathrm{pe}}$ /MPa
N1	1395	56.7	120.2	14.3	24.9	65.4	1113.5
N2	1395	56.7	120.2	14.3	24.9	65.4	1113.5
N3	1395	56.7	120.2	14.3	24.9	65.4	1113.5
N4	1395	33.6	92.7	14.9	30.7	68.4	1154.7

**Table 7:** Comparison of the experimental and predicted results of the undamaged box girder.

Bending Moment	Theoretical	Finite Element Analysis		Destructive Test	
	Value/kN·m	Value/kN·m	Ratio	Value/(kN·m)	Ratio
Cracking moment	6984	7272	1.04	7034	1.01
Ultimate bending moment	8442	13,831	1.64	13,500	1.60

**Table 8:** Calculation parameters of normal section flexural capacity of the damaged box girder.

Member	*ƒ*_cd_/MPa	*ƒ*_sd_/MPa	*ƒ*′_sd_/MPa	*ƒ*_pd_/MPa	*A*_s_/mm^2^	*A*′_s_/mm^2^	*A*_p_/mm^2^	*h*_0_/mm	*a*′_s_/mm	*x*/mm
Damaged box girder	22.4	280	280	1260	1140	2670	3475	1213	45	88.2

**Table 9:** Comparison of experimental and predicted values of damaged box girder with theoretical value.

Bending Moment	Theoretical	Finite Element Analysis		Destructive Test	
	Value/(kN·m)	Value/(kN·m)	Ratio	Value/(kN·m)	Ratio
Ultimate bending moment	5472	9657	1.76	8994	1.64

**Table 10:** Safety coefficient for different types of trucks.

Type of Truck	Action Effect		Safety Coefficient			
			Ultimate Bearing Capacity of the Undamaged Box Girder		Ultimate Bearing Capacity of the Damaged Box Girder	
	Bending Moment of the Dead Load	Bending Moment of Different Types of Trucks	Theoretical Prediction	According to The Destructive Test	Theoretical Prediction	According to the Destructive Test
	/kN·m	/kN·m	(8442 kN·m)	(13,500 kN·m)	(5472 kN·m)	(8994 kN·m)
1	2053	1720	3.71	6.66	1.99	4.04
2	2053	2301	2.78	4.97	1.48	3.02
3	2053	2947	2.17	3.88	1.16	2.36

## Data Availability

No new data were created or analyzed in this study. Data sharing is not applicable to this article.
